# Identification of New Sphingomyelinases D in Pathogenic Fungi and Other Pathogenic Organisms

**DOI:** 10.1371/journal.pone.0079240

**Published:** 2013-11-01

**Authors:** Camila Dias-Lopes, Izabella A. P. Neshich, Goran Neshich, José Miguel Ortega, Claude Granier, Carlos Chávez-Olortegui, Franck Molina, Liza Felicori

**Affiliations:** 1 Departamento de Bioquímica-Imunologia, Instituto de Ciências Biológicas, Universidade Federal de Minas Gerais, Belo Horizonte, MG, Brazil; 2 Centro de Biologia Molecular e Engenharia Genética, Universidade Estadual de Campinas (UNICAMP), Campinas, SP, Brazil; 3 Embrapa Information Technologies, Campinas, SP, Brazil; 4 SysDiag UMR 3145 CNRS/BioRad, Cap Delta, Montpellier, France; Russian Academy of Sciences, Institute for Biological Instrumentation, Russian Federation

## Abstract

Sphingomyelinases D (SMases D) or dermonecrotic toxins are well characterized in *Loxosceles* spider venoms and have been described in some strains of pathogenic microorganisms, such as *Corynebacterium* sp. After spider bites, the SMase D molecules cause skin necrosis and occasional severe systemic manifestations, such as acute renal failure. In this paper, we identified new SMase D amino acid sequences from various organisms belonging to 24 distinct genera, of which, 19 are new. These SMases D share a conserved active site and a C-terminal motif. We suggest that the C-terminal tail is responsible for stabilizing the entire internal structure of the SMase D Tim barrel and that it can be considered an SMase D hallmark in combination with the amino acid residues from the active site. Most of these enzyme sequences were discovered from fungi and the SMase D activity was experimentally confirmed in the fungus *Aspergillus flavus*. Because most of these novel SMases D are from organisms that are endowed with pathogenic properties similar to those evoked by these enzymes alone, they might be associated with their pathogenic mechanisms.

## Introduction

Sphingomyelinases D (SMases D; EC 3.1.4.41), also known as sphingomyelin phosphodieasterases D and sometimes as phospholipase D (PLD) [1], catalyze the hydrolytic cleavage of sphingomyelin or many other lysophospholipids to produce choline and ceramide 1-phosphate or choline and lysophosphatidic acid (LPA), respectively [2,3]. These enzymes are found in the venom of spiders from the Sicariidae family and are responsible for the outcome of local dermonecrosis in victims that suffer spider bites, as well as the systemic toxic effects, such as kidney failure and death [4–8]. Proteins with SMase D activity have been identified in arachnids, in the saliva of a tick from the genus *Ixodes* [9] and in a few strains of *Corynebacteria* and *Arcanobacterium* [10]. Recently, BLAST searches, though not accompanied with corresponding experimental evidence, have revealed the presence of homologous enzymes in the fungi *Aspergillus* and *Coccidioides* (UniProt accession numbers Q2UAL9, Q2UKE8, Q2U8X2 and Q1DU31) [11]. Intrigued by the presence of this toxic enzyme in medically important but distantly related organisms, such as spiders and bacteria, Cordes and Binford [12] identified a common motif at the C-terminal end of SMase D (without known function), supporting their inference about the origins of these enzymes from the broadly conserved glycerophosphoryl diester phosphodiesterase (GDPD; EC 3.1.4.46) family, in which, however, this motif is absent. 

In the present work, using a bioinformatics sequence similarity search methodology, we identified several new SMases D in different pathogenic organisms, such as spiders, bacteria, ticks, mites and fungi. A significant number of pathogenic fungal species were found to contain SMase D-like sequences, presenting strictly conserved catalytically critical amino acids. Thus, for the first time, an SMase D activity was experimentally demonstrated in a fungi. We also infer the function of the C-terminal conserved motif (SMD-tail) in stabilizing the entire internal structure of the SMase D TIM barrel. This work suggests that SMases D are widely represented in several genera and may act as a common pathogenic effector for a significant diversity of organisms. 

## Methods

### SMase D sequence similarity search and ortholog identification

A bidirectional best hit (BBH) approach for automated protein sequence similarity searches was performed using the SMase D protein sequences from *Loxosceles laeta* (GI: 60594084) and *Corynebacterium pseudotuberculosis* (GI: 300857446) as queries. The searches started with 5 iterations of PSI-BLAST [13] against a downloaded NCBI nr protein database (release number?). The sequences found as hits at the 5^th^ iteration (where the e-value was set to be better than the 1×10^-5^ threshold) were selected for further examination. To avoid misinterpretation of the PSI-BLAST results, the bidirectional best hit function was used to carefully select only the true positives. The protein sequences found as hits were used as queries in a BLASTp search against a test set database containing only the true positive (SMases D from *Corynebacterium, Loxosceles* and *Ixodes*) and true negative sequences (paralogs such as GDPD from diverse organisms) – both recovered from the BRENDA databank [14]. The protein sequences that matched the true positive sequences as best hits were classified as true orthologs and those that matched true negatives were not considered for further analysis. The above described search was not restricted only to the protein databases. In fact, a tBLASTn against the nucleotide database, which does not have a direct mapping to protein databases, was also performed. The subject databases chosen were: NCBI WGS (Whole Genome Shotgun sequences http://www.ncbi.nlm.nih.gov/genbank/wgs), NCBI dbEST (database of Expressed Sequence Tags http://www.ncbi.nlm.nih.gov/dbEST/) and NCBI TSA (Transcriptome Shotgun Assembly http://www.ncbi.nlm.nih.gov/genbank/tsa), all of which were downloaded from the NCBI ftp (ftp://ftp.ncbi.nih.gov/). The hits found were also checked using BLASTx against the test set database to identify the true positive hits. 

### Sequence analysis

One representative sequence for each genus was selected based on the following requirements: the confirmed existence of either structural or experimental data linking the protein sequence to SMase D activity, the confirmed existence of the complete protein sequence deposited at the corresponding NCBI hosted database, the confirmed existence of a complete nucleotide sequence deposited in WGS or other NCBI nucleotide database and finally the verifiable possibility to build contigs using cap3 [15] for the entire or major part of the coding sequence from transcriptome databases. Constrained-based multiple alignments were performed using COBALT [16]. Alignment analyses and editing were performed using Jalview 2.5 [17]. 

The phylogenetic analysis was performed using one representative putative SMase D sequence for each genus. Clustal W was used for the multiple sequence alignments and MEGA 4 [18] for the creation of Maximum Likelihood phylogenetic trees. The Jones, Taylor and Thornton (JTT) model [19] and the γ-based method were used for correcting the heterogeneity rates among sites. Bootstrap replicates (1000) were calculated to access the confidence of the tree.

### SMase D structural analysis

To gain insights about the differences and similarities of the enzymes from the different organisms, structural bioinformatics analyses were performed. First, the 3D structures of *Corynebacterium pseudotuberculosis* and *Aspergillus flavus* SMase D (sequence GIs: 300857446 and 220691453) were predicted and comparisons of the generated models with the crystallographic structure of SMase D 1 from *Loxosceles laeta* were performed with previously predicting secondary structure elements in the target sequences using Jpred [20]. Homology models were built using the YASARA molecular modeling package [21]. For all modeling, the “hm_build” macro of the YASARA package was used with the default parameters except for the oligomerization state, which was set to “monomeric”. The crystal structure of *L. laeta* (PDB code 1XX1) was used as a template. The models were initially refined using YASARA and the system containing a protein immersed in a water box was optimized using energy minimization to remove any steric clashes and, later, using the molecular dynamics YASARA macro (“md_run”) during 1ns. The default simulation parameters were maintained at the values defined by the macro. The simulation used the AMBER03 force field. The modeled structures were then examined and the best representative structure was chosen based on the calculated energy of the structure by YASARA. The quality of the final model was also checked using Prosa-Web [22], space-clash analysis and Ramachandran plot analysis using STING Java Protein Dossier [23,24]. Structural alignments were calculated using the software MUSTANG [25]. SMase I (PDB code 1XX1) image rendering was performed using the STING Java Protein Dossier, SwissPDB-Viewer package [26] and the PyMOL Molecular Graphics System, Version 1.5.0.4 Schrödinger, LLC.

 The identification of contacts for the SMase D (PDB code “1xx1.pdb”) was obtained using the STING Java Protein Dossier and Sting Report [23,24,27] and its STING Contacts module [28]. Protein structure images were created using the PyMOL Molecular Graphics System, Version 1.5.0.4 Schrödinger, LLC. Data regarding contact type identification, atoms and the residues involved were stored and analyzed in a MySQL database. The secondary structures were defined by STING module SS_PDB, SS_STRIDE and SS_DSSP for *L. laeta* SMases D. For the modeled structures of *C. pseudotuberculosis* and *A. flavus*, the secondary structure elements were defined by STING module SS_Stride. Loop positions and extensions as well as the positions of the secondary structure elements at spider SMases D were defined based on the position of the first residue after a defined helix or strand and up to the last residue before a new helix or strand. For the analysis, we only considered loops containing more than 3 residues and when two of the three secondary structure predictors (SS_PDB, SS_STRIDE and SS_DSSP) agreed about the position of the secondary structure element after and before the loop. Graphics of the secondary structure element positions were generated using the GraphPad Prism 5 software.

The process of defining the active site nanoenvironment was performed by selecting a set of STING physicochemical and structural parameters that uniquely characterized the most important residues for the SMase D catalytic activity. A construct “nanoenvironment” was previously defined in our earlier work regarding the characterization for the specificity of ligand binding, protein function preservation in mutants and the specificity of enzymes based on the type of interface residues (Fernandez et al., 2003 [29]; Marcellino et al., 1996 [30] and Ribeiro et al., 2010 [31], respectively). As defined by Murakami et al. (2005)[2], His12 and His47 are the key catalytic residues, followed by the metal ion coordinating residues Glu32, Asp34 and Asp91. Using the Select tool of the STING Java Protein Dossier [23], the active site was characterized using a set of parameters that was subsequently tested for the structure of spider SMase D class II (PDB code 3RLH) and the modeled structures of the fungal and bacterial SMases D. 

### SMase D activity measurement

SMase D activity was measured using the Amplex Red Sphingomyelinase kit (Invitrogen, Paisley, UK) following the manufacturer’s instructions, however; alkaline phosphatase was omitted to quantify only the SMase D-specific activity. Fluorescence emissions were measured using an Infinite F200 spectrophotometer (Tecan, Männedorf, Switzerland). *Aspergillus flavus* extract were obtained by filtration of cultures after 10 days incubation at 30 °C from Bio-Rad Laboratories (Reference code: 52942C1).

## Results

### Identification of new SMases D in different organisms

**Figure 1:**
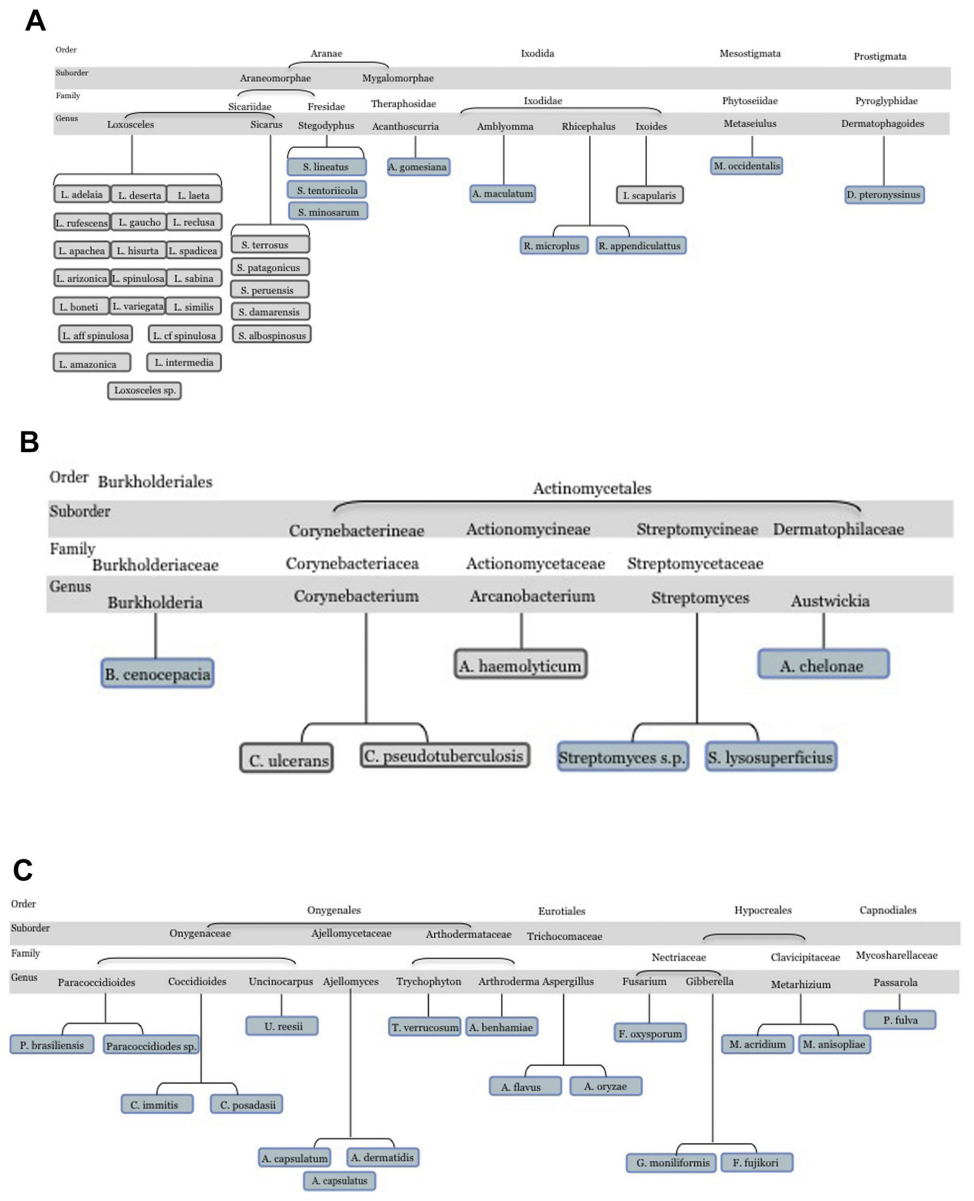
Organisms with SMase D-like proteins. Metazoa (A), bacteria (B) and fungi (C). The species in gray have been described as possessing SMase D activities in previous works and, in blue, are the new species with SMase D-like proteins found in this work.

Bidirectional best hits (BBH) analyses were performed to identify candidate SMase D enzymes in different species. This strategy was able to identify SMase D-like sequences in 24 distinct genera, 59 species and 105 subspecies upon extended searches in different databases such as NCBI nr, WGS, dbEST and TSA. In addition, the incomplete sequence of the *Acari* from the genera *Dermatophagoides, Varroa, Psoroptes* and *Tetranychus* were identified as possible SMase D-like proteins (data not shown). As shown in Figure 1, a significant variety of fungi ((Figure 1C, 11 genera) contain SMase D-like sequences compared to bacteria (Figure 1B), spiders and *Acari* (Figure 1A) (5, 4 and 4 genera, respectively). The majority of these sequences have never before been identified *in silico* or *in vitro*. For the 5 bacterial genera found, 3 were new: *Burkholderia, Streptomyces* and *Austwickia* (Figure 1 B, blue squares). Surprisingly, from the Sicariidae family, 2 new spider genera were also found: *Acanthoscurria* and *Stegodyphus* (Figure 1A), in which SMase D occurrence has never been reported until now. The searches identified SMase D similar sequences in 3 different *Acari* genera besides *Ixodes scapularis* (Figure 1A), where SMase D activity has been previously reported [9]. The novel *Acari* genera found were: *Amblyomma*, *Rhipicephalus* and *Metaseiulus*. In regards to fungi, the genera found were: *Ajellomyces* or *Histoplasma*, *Arthroderma*, *Trichophyton*, *Uncinocarpus*, *Coccidioides*, *Paracoccidioides*, *Aspergillus*, *Gibberella*, *Fusarium*, *Metarrhizium* and *Passalora*. The complete list of species, as well as the database source of SMase D-like sequences, is shown in Tables S1, S2 and S3.

### Newly discovered SMases D share a conserved active site and C-terminal motif

**Figure 2:**
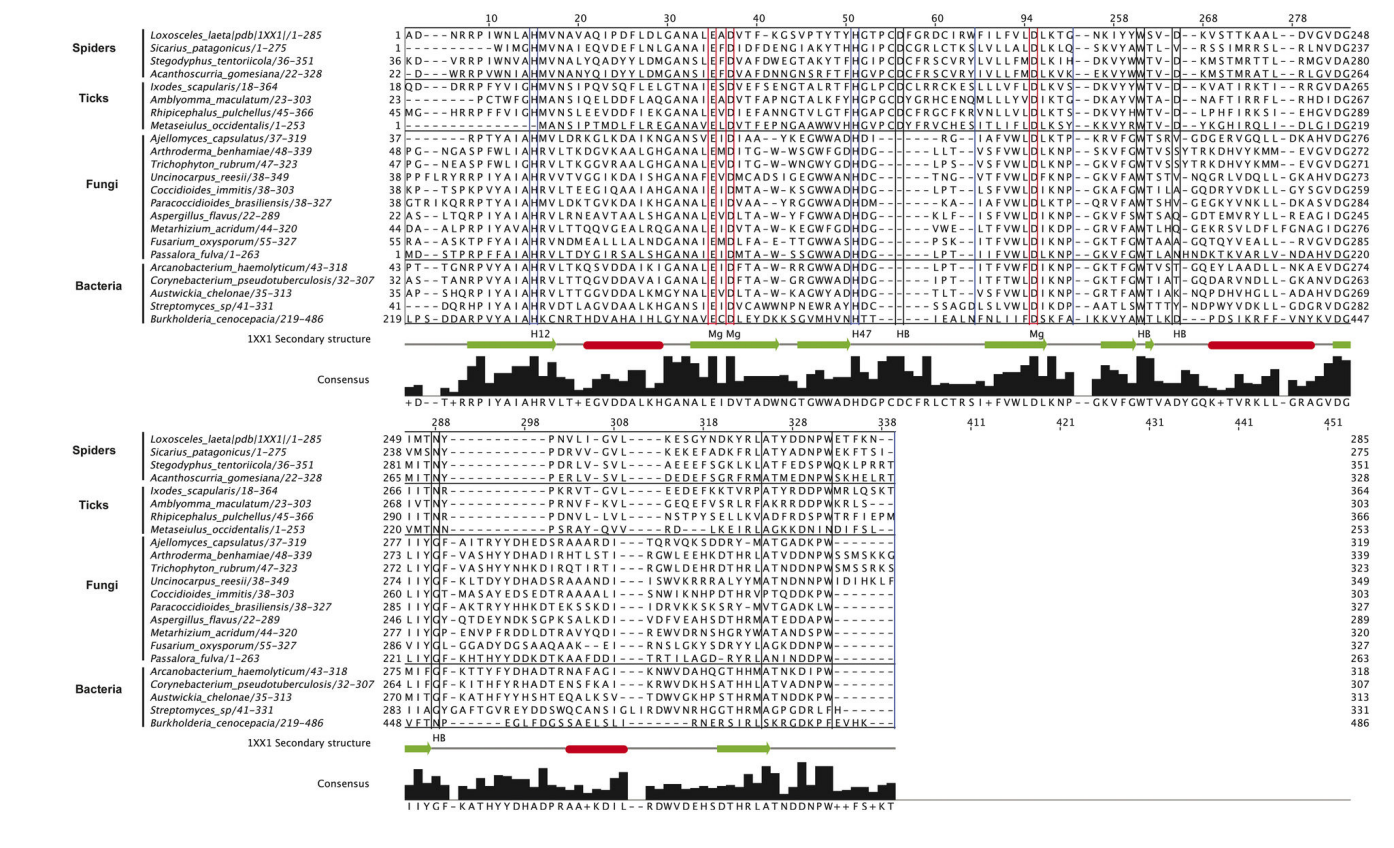
Multiple sequence alignment of one SMase D sequence for each genus identified. Consensus sequences and secondary structure of *L. laeta* SMase D (pdb number:1xx1) are shown underneath the alignment. Conserved catalytic histidines residues are marked with blue boxes (Label H12 and H47 in secondary structure of 1xx1). Residues responsible for Mg^+2^ coordination are marked with red boxes (Label Mg in secondary structure of 1xx1) and amino acids involved in network of hydrogen bonds are marked with black boxes (label HB in secondary structure of 1xx1). The following sequences were used to build this alignment and were obtained from the NCBI nr protein database: *Ajellomyces* (GI: 225556466), *Arthroderma* (GI: 302499957), *Trichophyton* (GI: 327309460), *Uncinocarpus* (GI: 258566630), *Coccidioides* (GI: 119182286), *Paracoccidioides* (GI: 225681750), *Aspergillus* (GI: 220691453), *Metaseiulus* (GI: 391334832), *Loxosceles* (GI: 60594084), *Sicarius* (GI: 292630576), *Ixodes* (GI: 121962650), *Amblyomma* (GI: 346467405), *Rhipicephalus* (GI: 427782159), *Burkholderia* (GI: 116687281), *Arcanobacterium* (GI: 297571658), *Corynebacterium* (GI: 300857446), Streptomyces (GI: 254384004) and *Austwickia* (GI:403190737). Six genera have not entries in NCBI nr protein database, but their protein sequences were translated from in NCBI WGS or dbEST entries:, *Fusarium* (GI: 144921672, position: 31242-32224), *Gibberella* (GI: 116139506, position: 93710-94513), *Passalora* (GI: 407486978, position: 4015-4803) , *Metarhizium* (GI: 322696462), *Stegodyphus* (GI: 374247203) and *Acanthoscurria* (GIs: 68762186 and 68761727). The genera *Dermatophagoides*, *Psoroptes*, *Varroa* and *Tetranycus* have incomplete sequences and were not used in this analysis.

Structural studies on SMase I (PDB 1XX1), an SMase D from *Loxosceles laeta* [2], have suggested that the catalytic mechanism of this eight-stranded TIM barrel (or α/β barrel) SMase D involves an acid-base reaction that requires two histidine residues (His12 and His47) and the presence of one Mg^+2^ ion. The alignment (Figure 2) of one sequence of each genera showed that the catalytic histidine residues (12 and 47) are strictly conserved in all the newly identified sequences, except for the mite *Metaseiulus occidentalis*, which lacks His12. The residues Glu32, Asp34 and Asp91, which coordinate the Mg^+2^ ion, are also strictly conserved. In this family of proteins, the catalytic His12 and His47 are supported by a network of hydrogen bonds between Asp34, Asp52, Trp230, Asp233 and Asn252. Asp34 and Trp230 are strictly conserved in the aligned sequences of Figure 2; however, Asp52, Asp233 and Asn252 are not conserved. 

**Figure 3:**
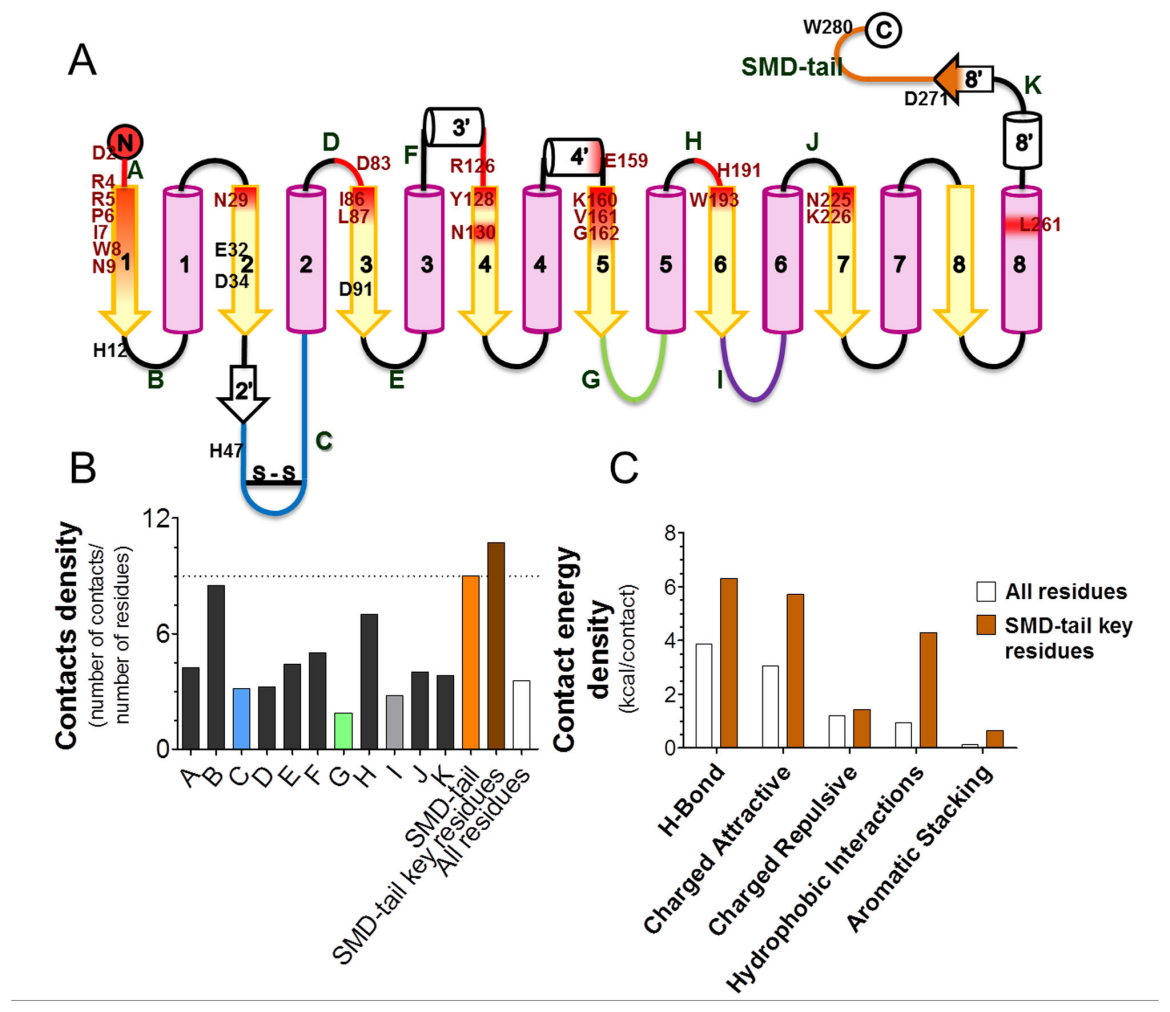
SMD-tail contacts with inner β-strands and loop contact densities in the *L. laeta* SMase D. (A) Scheme for SMase D secondary structure elements, as described in Murakami et al. (2005) and indicating the SMD-tail (orange), the catalytic, flexible and variable loops (shown in blue, green and purple, respectively), the inner β-strands (yellow) and α-helices (light pink) numbered from 1 to 8. The position of the interaction partners of the SMD-tail (shown in red) and the loops with more than 3 residues coded from A to K. (B) Contact density values obtained by STING contacts for each loop and the entire SMD-tail, the SMD-tail key residues (271-274, 277, 279 and 280) and the ratio for all residues in the protein. (C) Comparison of contact energy density values for the SMD-tail to the entire protein and each type of contact.

It has previously been reported that, along with the fairly well conserved active site, SMases D have a conserved C-terminal motif (without, as of now, any known function) that distinguishes them from the GDPD enzyme family. This SMD-tail, which is located after the last α-helix of the TIM barrel in the C-terminus of SMase D, contains the ATXXDNPW motif, which is well conserved in most of the sequences (Figure 2). Due to its strong conservation, the SMD-tail could be expected to play an important functional or structural role. By analyzing the *L. laeta* SMase D (PDB 1XX1, chain A) structure using the STING Java Protein Dossier, we found several pieces of evidence indicating the involvement of the SMD-tail in SMase D structure stabilization. Namely, these residues establish a significant number of contacts directly with residues located at seven (out of eight) inner β-strands from the TIM barrel structure, all being on the side of the protein opposite to the catalytic site. The two residues that established the energetically strongest contacts were Asp277 and Trp280. Trp280, as predicted by STING (Figure S1), establishes a hydrogen bond network with Glu159, Asn225 and Asn278 that is mediated by two water molecules. There was a nearly parallel aromatic stacking with His191 and another stacking with residue Tyr128. Hydrophobic contacts were predicted involving Trp193, Gly162, Val161, Lys160, Val130 and Tyr128. Asp277, another highly conserved residue in the SMD-tail motif, also establishes important contacts, predominantly ionic interactions with Arg271, a hydrogen bond (intermediated by two water molecules) with Trp8 from the N-terminal β-sheet hydrogen bonds with Pro279 and Thr274 and hydrophobic contacts with Pro6 (Figure S1). Thus, the conservation of the SMD-tail throughout the family is possibly due to its significant importance in the structural stabilization of inner barrel β-sheets. To strengthen this hypothesis, the contact density and energies established among the enzyme residues were calculated. Both the density of the contacts and the contact energy density are higher in the SMD-tail compared to the entire protein (e.g., 10.7 *versus* 3.57, as shown in Figure 3B). When compared to all SMase D loops, the SMD-tail is still better with regards to the contact density (Figure 3B). However, the B loop, which contains the catalytic His12, also had a high contact ratio. Considering the distinct types of contacts, there was an average of 2.4 hydrogen bonds *per* residue in the SMD-tail, whereas this value is much lower when the entire protein was considered (1.5), as shown in Figure 3C. A similar trend was observed for charged attractive and hydrophobic interactions. 

### Analysis of fungal and bacterial SMases D reveals a conserved catalytic site nanoenvironment

**Figure 4:**
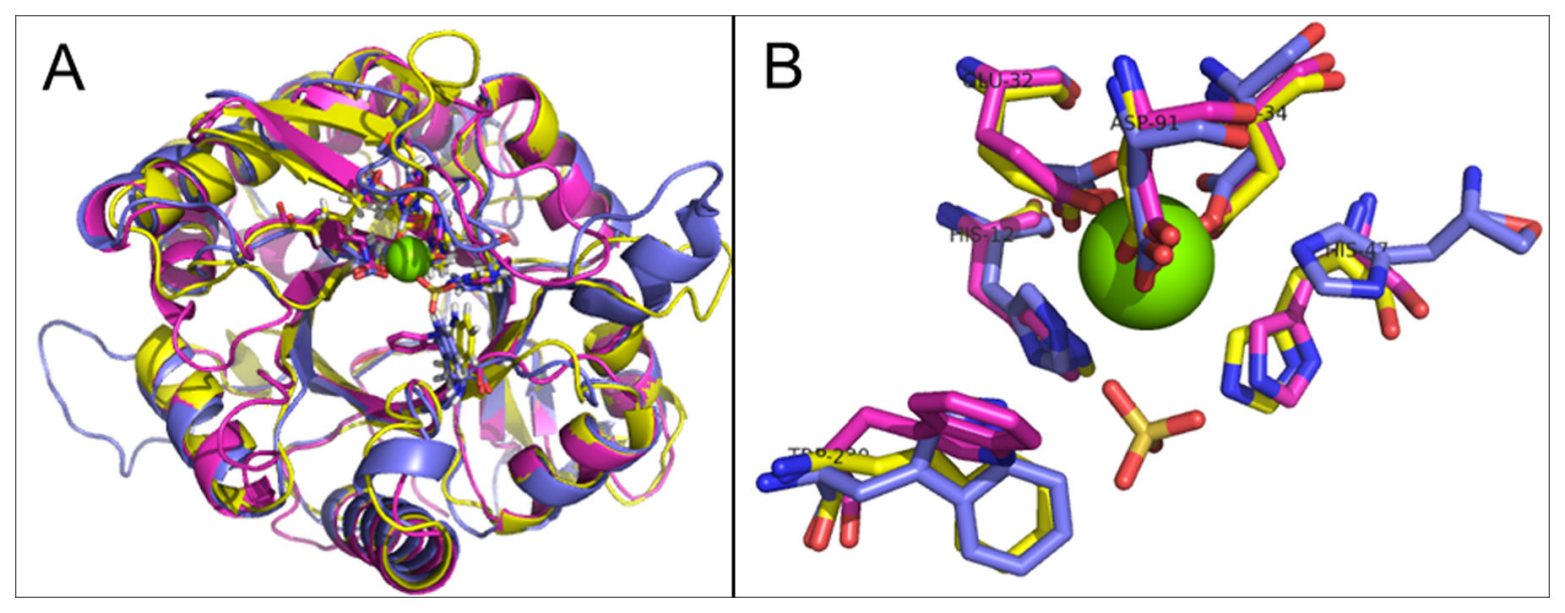
Structural alignment of SMases D. *L. laeta* SMase D (1xx1) (purple) and the models for the SMase D-like proteins from the fungus *A. flavus* (yellow) and from *C. pseudotuberculosis* (blue), indicating the overall alignment (A) and the active site residues superposed in sticks (B), the Mg^+2^ ion as a green sphere and the co-crystalized SO^4-^ ion in sticks.

Although the primary sequence similarity of the *Aspergillus* and *Corynebacteria* SMases D to the template structure *L. laeta* SMase D is very low (22% and 20%, respectively), the secondary structure prediction suggested a significant structural overlap among the proteins from fungi, bacteria and spiders, allowing for the construction of models with good structural indicators. The produced models were structurally aligned to the template *L. laeta* SMase D (PDB code “1xx1.pdb”), producing a RMSD of 0.79 (249 Cα aligned) and 1.018 Å (over 225 Cα aligned), respectively, for the *Aspergillus flavus* and *Corynebacterium pseudotuberculosis* SMases D (Figure 4A). The active site residues, His12, His47, Glu32, Asp34 and Asp91, are readily superimposable (Figure 4B). In addition, using the STING Java Protein dossier parameters, similar physicochemical and structural features were identified in the bacterial, fungal and spider SMases D proteins (Figure S1). 

### *In vitro* evaluation of the SMase D activity in *Aspergillus flavus*

**Figure 5:**
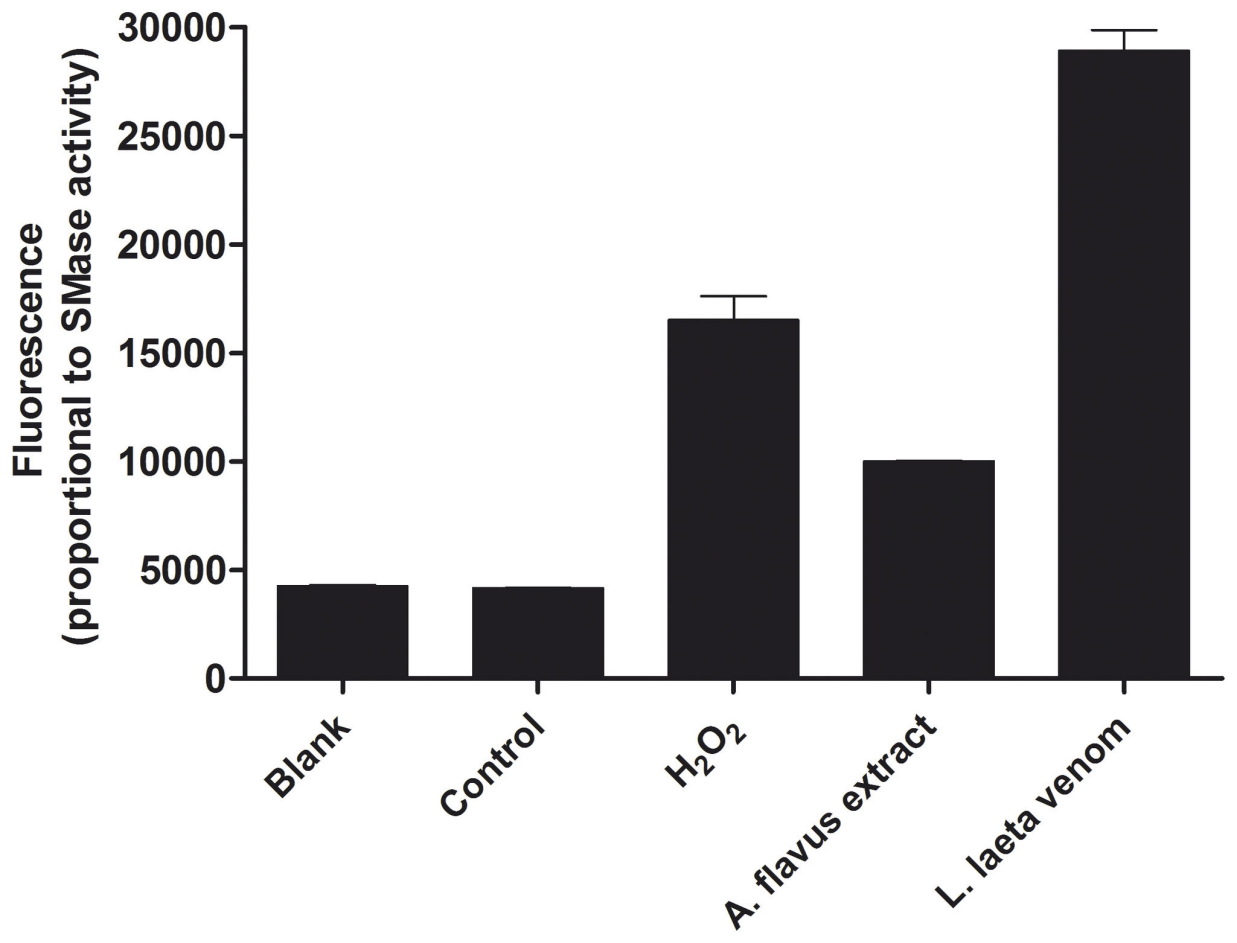
Fluorescence intensity (proportional to the SMase D activity) using 0.5 µg of *Loxosceles* *laeta* crude venom and 50 µg of *Aspergillus flavus* extract. H_2_O_2_ was used as a positive control, whereas the negative controls were SMase C from *Bacillus cereus* (control) and reaction buffer alone (blank).

With the aim of verifying if one of the newly identified SMase D proteins exhibits SMase D activity, we tested the enzymatic activity of *A. flavus* extracts and compared it to that of *L. laeta* crude venom, which is known to possess a high SMase D activity. Indeed, the *A. flavus* extract could hydrolyze sphingomyelin, producing a measureable fluorescence (Figure 5). The lower SMase D activity of *A. flavus* compared to the *L. laeta* venom can be explained by the lower concentration of this enzyme in the *A. flavus* extract and/or by a lower specific enzymatic activity. Moreover, the SMase D activity of *A. flavus* extracts was reduced by 33% following the addition of 10 mM EDTA, consistent with the known ion-dependent catalytic mechanism of SMase D (data not shown).

### Phylogenetic analysis of SMases D

**Figure 6:**
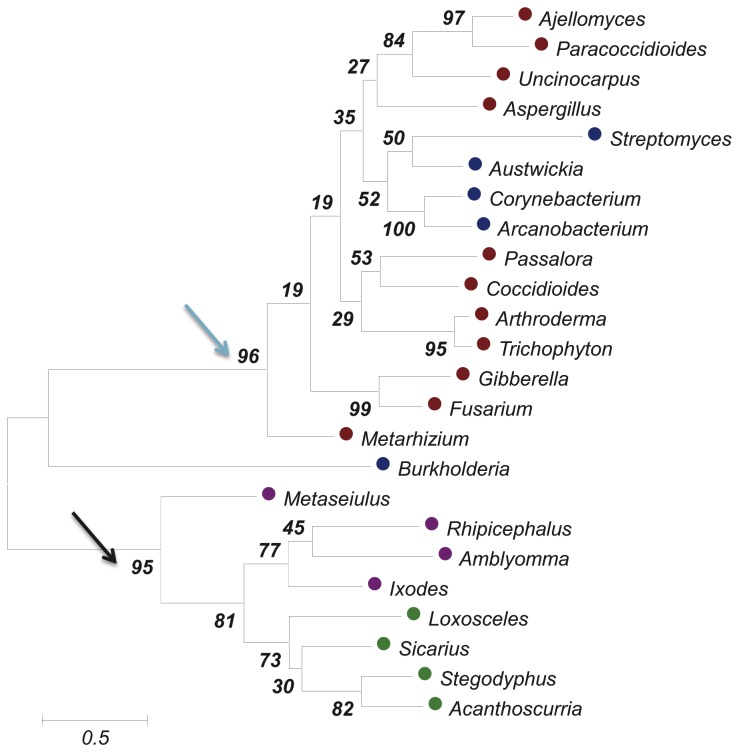
Phylogenetic analysis of SMase D-like sequences from spiders, ticks, bacteria and fungi. The circles indicate the clade of each species (red for fungi, blue for bacteria, purple for ticks and green for spiders). The arrows indicate that the high bootstrap values suggest high levels of confidence for grouping the spider and tick proteins together into the same clade (black arrow), as well as the bacteria and fungi (blue arrow). The following sequences were used to build this phylogenetic tree and were obtained from the NCBI nr protein database: *Ajellomyces* (GI: 225556466), *Arthroderma* (GI: 302499957), *Trichophyton* (GI: 327309460), *Uncinocarpus* (GI: 258566630), *Coccidioides* (GI: 119182286), *Paracoccidioides* (GI: 225681750), *Aspergillus* (GI: 220691453), *Metaseiulus* (GI: 391334832), *Loxosceles* (GI: 60594084), *Sicarius* (GI: 292630576), *Ixodes* (GI: 121962650), *Amblyomma* (GI: 346467405), *Rhipicephalus* (GI: 427782159), *Burkholderia* (GI: 116687281), *Arcanobacterium* (GI: 297571658), *Corynebacterium* (GI: 300857446), *Streptomyces* (GI: 254384004) and *Austwickia* (GI:403190737). Six genera have no entries in the NCBI nr protein database, but their protein sequences were translated from NCBI WGS or dbEST entries: *Histoplasma* (GI: 421925211, position: 21238-22200), *Fusarium* (GI: 144921672, position: 31242-32224), *Gibberella* (GI: 116139506, position: 93710-94513), *Passalora* (GI: 407486978, position: 4015-4803), *Metarhizium* (GI: 322696462), *Stegodyphus* (GI: 374247203) and *Acanthoscurria* (GIs: 68762186 and 68761727). The genera *Dermatophagoides*, *Psoroptes*, *Varroa* and *Tetranycus* have incomplete sequences and were not used in this analysis.

Phylogenetic analysis of the SMase D-like sequences for one representative sequence of each genus clearly placed the spider and Corynebacterineae proteins into distinct clades. In addition, the bacterial proteins grouped together with the fungal proteins of Pezizomycotina fungi with a high bootstrap value (Figure 6). It is possible that lateral gene transfer (LGT) events occurred “recently” among fungal and bacterial species, resulting in highly conserved SMase D sequences, reaching 60% similarity. The bacterial species were found interspersed within the fungi clade but not necessarily forming a monophyletic clade. Another interesting consideration here is that a number of genomes were sequenced from the same suborders of *Corynebacterium* (Corynebacterineae, 244 genome sequences), *Streptomyces* (Streptomycineae, 69 genome sequences), *Arcanobacterium* (Actinomycineae, 33 genome sequences) and *Austwickia* (Micrococcineae, 148 sequences) and SMase D-like sequences were only found in these four genera (for the species and strains shown in table S1). Finally, the phylogenetic analysis showed that SMases D possibly evolved by convergence independently from a GDPD ancestor in arthropods and in fungi. 

## Discussion

Phopholipases are enzymes found in many organisms and have various functional roles. They constitute a heterogeneous group of enzymes described as essential virulence or toxic factors, including phospholipases A (PLA)[32], B (PLB) [33], C (PLC) [34] and D (PLD) [35,36]. Among the PLD group, a unique class of enzymes, commonly known as sphingomyelinase D (SMase D), shows no sequence or structural homology with other phospholipid-metabolizing enzymes. They lack the conserved HKD sequence motif that characterizes the PLD superfamily. SMase D activity has been reported previously in *Loxosceles* and *Sicarius* spiders [37], in *Corynebacterium* and *Arcanobacterium* bacteria [10] and in the tick *Ixodes scapularis* [9]. 

In this work, after extensive database searches of proteomes, whole genomes and transcriptome assemblies, it was possible to locate a significant number of organisms that contain genes similar to *Corynebacterium pseudotuberculosis* and *Loxosceles laeta* SMase D genes. By analyzing the SMase D sequence of the 24 genera found in this work, we observed a strict conservation of the catalytic His12 and His47 and residues Glu32, Asp34 and Asp91, which coordinate the Mg^+2^ ion. In this family of proteins, the catalytic residues are supported by a network of hydrogen bonds between Asp34, Asp52, Trp230, Asp233 and Asn252. Asp34 and Trp230 are strictly conserved; however, Asp52, Asp233 and Asn252 are not conserved (Figure 2). There was no consensus among these amino acids in the different SMase D-like proteins of the different organisms, likely because the hydrogen bond contribution of the carbonyl groups of these amino acids can be replaced by any other donor/acceptor group. In addition, sequence analyses revealed a highly conserved C-terminal region establishing a significant number of contacts directly with residues located at seven (out of eight) inner β-strands from the TIM barrel structure. This finding suggests that the C-terminal SMD-tail motif not only “plugs” the N-terminal end of the barrel, as suggested by Cordes and Binford, 2006 [12], but also acts as a structural stabilizer of the entire structure of the TIM barrel. It is also possible that the SMD-tail is involved in the establishment of functional interactions. We suggest here that the SMD-tail can be considered an SMase D hallmark in combination with residues from the conserved active site.

Among such newly identified organisms, we could locate a significant number of pathogenic fungal species for which there were no previous records in the literature regarding the presence of an SMase D, with the exception of BLAST searches indicating homologous enzymes in some fungi (*Aspergillus* and *Coccidioides* UniProt accession numbers: Q2UAL9, Q2UKE8, Q2U8X2 and Q1DU31) [11], without experimental evidence. We experimentally verified the occurrence of SMase D enzymatic activity in *Aspergillus flavus* extract.

*Aspergillus flavus* is one of the main species that causes human invasive aspergillosis, characterized by the invasion and necrosis of lung parenchyma by *Aspergillus* [38]. It is also the main agent of acute and chronic invasive and granulomatous *Aspergillus* sinusitis, an agent of otitis and keratitis. The presence of SMase D activity in this organism could have a role in the inflammatory process of the respiratory tract and in invasion and necrosis. In addition to *Aspergillus*, most of the new fungi described in this work presenting SMase D activity have been described as pathogens involved in cutaneous infections, such as *Tricophyton* and *Arthroderma* [39] and cutaneous infections as well as necrosis and pulmonary infections, such as *Histoplasma, Paracoccidioidis* and *Coccidiodis* [40–43]. The newly described genera of ticks, such as *Rhipicephalus* and *Amblyomma*, are involved in animal ear inflammation, possibly necrotic [44–46]. *Streptomyces* can cause infections in humans, such as mycetome [47] and *Burkholderia cenocepacia* is described as being involved in “cepacia syndrome”, characterized by, among others symptoms, uncontrolled bronchopneumonia [48]. In addition, some fungi identified in this work with SMase D activities are plant pathogens, such as *Gibberela, Fusarium, Metarhizium* and *Passalora* [49–51].

As can be observed, most of the new organisms identified and having a potential SMase D activity in this work are human, animal or plant pathogens. 

Are SMase D enzymes a toxic factor common to these organisms and important to their pathogenicity? 

SMases D are found in the venom of spiders of the Sicariidae family and are responsible on their own for the local and also for the systemic, toxic effects following envenomation [52] . In addition, they are by themselves capable of provoking characteristic necrotic skin lesions [4]. In *Archanobacteria* and *Corynebacteria*, SMase D activity is a potent virulence factor functioning to trigger hemolysis and vascular permeabilization. This activity allows the bacteria, which infects via skin abrasions, to move into the host lymph nodes, where the infection localizes. Within the lymph nodes, the bacteria invade macrophages and replicate intracellularly. Ceramide-1-phosphate generation has been proposed to remodel lipid rafts within the outer leaflet of the plasma membrane, concentrating lipid raft-bound proteins and receptors, thereby enhancing protein-mediated bacterial adhesion to the macrophage plasma membrane and host cell death following bacterial invasion [1,52]. SMase D deletion strains exhibit the decreased intracellular release of bacteria from the membrane-bound vacuole, suggesting that SMase D might also trigger vacuole membrane disruption [1,52]. 

This family of enzymes is described as producing choline to generate ceramide-1-phosphate from sphingomyelin (SM), profoundly altering the lateral structure and morphology of the target membrane. The SMase D also hydrolyzes lysophosphatidylcholine (LPC) to generate LPA [53]. LPA, a bioactive molecule that triggers a myriad of signaling cascades via G protein-coupled receptors (GPCR), is a known inducer of platelet aggregation and endothelial hyperpermeability and this activity is important for eliciting the inflammatory response (release of proinflammatory cytokines/chemokines) observed upon infection [54].

Considering the described effects elicited by SMase D enzymes in spiders and bacteria, SMase D, as a common enzyme in many pathogenic organisms, might play a role in pathogenicity through different mechanisms, such as membrane destabilization and host cell penetration [35] or host cell death following bacterial invasion [1] but also in pulmonary inflammation and cutaneous lesions, as described in spider and bacterial infections [52]. 

Finally, the phylogenetic analysis showed that SMases D possibly evolved by convergence independently from a GDPD ancestor in arthropods and in fungi. In bacteria, due to the significant similarity to fungal sequences and its presence in few species from terminal nodes (e.g., only two from the dozens of known *Corynebacterium*), it is possible that “recent” lateral gene transfer events were responsible for introducing the gene into the above mentioned groups, such as *Corynebacterium*. These data are in disagreement with the previously discussed lateral gene transfer of the SMase D gene among *Loxosceles* and *Corynebacterium* but in accordance with Fry and coworkers [11,12]. If a LGT event, such as proposed by Cordes and Binford (2006) [12], occurred, one should expect that spiders and bacteria would group together in a ML tree; however, this does not occur. More studies should be conducted to explore the evolutionary puzzle of SMases D. 

In conclusion, this bioinformatics analysis has allowed for the identification of SMase D enzymes in nineteen new genera never described before, presenting conserved catalytic amino acids and a C-terminal region important for structural stabilization. Most of these enzyme sequences were discovered in fungi and the SMase D activity was experimentally confirmed in the fungus *Aspergillus flavus*. Because most of these novel SMases D are from organisms that are pathogenic, they might be associated with their pathogenic mechanisms. Further, the identification of SMases D in new genomes or proteomes may be predictive of a potential toxicity. These new findings provide additional entry points for more profound studies on the role, occurrence and evolution of SMases D.

## Supporting Information

Figure S1 **Analysis of the contact characteristics of the SMS-tail residues in the 3D structure of the *L. laeta* SMase D (1xx1.pdb) according to STING predicted contacts.** The carbon atoms for the stick-represented residues are colored based on the STING color codes for the contact types: green for salt bridges or a charge attractive interaction, red for a charge repulsive interaction, gray for aromatic stacking, purple for a hydrophobic interaction, light pink for hydrogen bonds involving the main chain, orange for hydrogen bonds involving the main chain and a side chain and white for the query/central residue. The contact partners and interaction type are shown for the SMS-tail residues in the PDB file (1xx1.pdb): Arg271 (A), Leu272 (B), Ala273 (C), Thr274 (D), Asp277 (E), Pro279 (F) and Trp280 (G). An overall view of the SMS-tail is shown (H). STING graphical contact representation for the most important residues in terms of contact energy: Asp277 (I) and Trp280 (J).
(DOCX)

Table S1 **Bacterial species found to contain an SMaseD, with the corresponding databases for the sequences indicated.**
(DOCX)

Table S2 **Arachnid species found to contain an SMaseD, with the corresponding database sources for the sequences indicated.**
(DOCX)

Table S3 **Fungal species found to contain an SMaseD, with the corresponding database sources for the sequences indicated.**
(DOCX)

## References

[1] Lucas EA, Billington SJ, Carlson P, McGee DJ, Jost BH (2010) Phospholipase D promotes *Arcanobacterium* *haemolyticum* adhesion via lipid raft remodeling and host cell death following bacterial invasion. BMC Microbiol 10: 270. doi:10.1186/1471-2180-10-270. PubMed: 20973961. 20973961 PMC2978216

[2] Murakami MT, Fernandes-Pedrosa MF, Tambourgi DV, Arni RK (2005) Structural basis for metal ion coordination and the catalytic mechanism of sphingomyelinases D. J Biol Chem 280: 13658–13664. doi:10.1074/jbc.M412437200. PubMed: 15654080. 15654080

[3] Lee S, Lynch KR (2005) Brown recluse spider (*Loxosceles* *reclusa*) venom phospholipase D (PLD) generates lysophosphatidic acid (LPA). Biochem J 391: 317–323. doi:10.1042/BJ20050043. PubMed: 15926888. 15926888 PMC1276930

[4] Tambourgi DV, Magnoli FC, Van Den Berg CW, Morgan BP, De Araujo PS et al. (1998) Sphingomyelinases in the venom of the spider *Loxosceles* *intermedia* are responsible for both dermonecrosis and complement-dependent hemolysis. Biochem Biophys Res Commun 251: 366–373. doi:10.1006/bbrc.1998.9474. PubMed: 9790962. 9790962

[5] Tambourgi DV, Morgan BP, De Andrade RM, Magnoli FC, Van Den Berg CW (2000) *Loxosceles* *intermedia* spider envenomation induces activation of an endogenous metalloproteinase, resulting in cleavage of glycophorins from the erythrocyte surface and facilitating complement-mediated lysis. Blood 95: 683–691. PubMed: 10627480. 10627480

[6] Tambourgi DV, De F, Fernandes Pedrosa M, Van Den Berg CW, Gonçalves-de-Andrade RM, Ferracini M et al. (2004) Molecular cloning, expression, function and immunoreactivities of members of a gene family of sphingomyelinases from *Loxosceles* venom glands. Mol Immunol 41: 831–840. doi:10.1016/j.molimm.2004.03.027. PubMed: 15234562. 15234562

[7] Chaim OM, Sade YB, Da Silveira RB, Toma L, Kalapothakis E et al. (2006) Brown spider dermonecrotic toxin directly induces nephrotoxicity. Toxicol Appl Pharmacol 211: 64–77. doi:10.1016/j.taap.2005.05.015. PubMed: 16005484. 16005484

[8] Kusma J, Chaim OM, Wille ACM, Ferrer VP, Sade YB et al. (2008) Nephrotoxicity caused by brown spider venom phospholipase-D (dermonecrotic toxin) depends on catalytic activity. Biochimie 90: 1722–1736. doi:10.1016/j.biochi.2008.07.011. PubMed: 18760322. 18760322

[9] Alarcon-Chaidez FJ, Boppana VD, Hagymasi AT, Adler AJ, Wikel SK (2009) A novel sphingomyelinase-like enzyme in Ixodes scapularis tick saliva drives host CD4 T cells to express IL-4. Parasite Immunol 31: 210–219. doi:10.1111/j.1365-3024.2009.01095.x. PubMed: 19292772. 19292772 PMC2748904

[10] McNamara PJ, Cuevas WA, Songer JG (1995) Toxic phospholipases D of *Corynebacterium* *pseudotuberculosis*, *C.* *ulcerans* and *Arcanobacterium* *haemolyticum*: cloning and sequence homology. Gene 156: 113–118. doi:10.1016/0378-1119(95)00002-N. PubMed: 7737503. 7737503

[11] Fry BG, Roelants K, Champagne DE, Scheib H, Tyndall JDA et al. (2009) The toxicogenomic multiverse: convergent recruitment of proteins into animal venoms. Annu Rev Genomics Hum Genet 10: 483–511. doi:10.1146/annurev.genom.9.081307.164356. PubMed: 19640225. 19640225

[12] Cordes MHJ, Binford GJ (2006) Lateral gene transfer of a dermonecrotic toxin between spiders and bacteria. Bioinformatics 22: 264–268. doi:10.1093/bioinformatics/bti811. PubMed: 16332712. 16332712

[13] Altschul SF, Madden TL, Schäffer AA, Zhang J, Zhang Z et al. (1997) Gapped BLAST and PSI-BLAST: a new generation of protein database search programs. Nucleic Acids Res 25: 3389–3402. doi:10.1093/nar/25.17.3389. PubMed: 9254694. 9254694 PMC146917

[14] Barthelmes J, Ebeling C, Chang A, Schomburg I, Schomburg D (2007) BRENDA, AMENDA and FRENDA: the enzyme information system in 2007. Nucleic Acids Res 35: D511–D514. doi:10.1093/nar/gkl972. PubMed: 17202167. 17202167 PMC1899097

[15] Huang X, Madan A (1999) CAP3: A DNA Sequence Assembly Program. Genome Res 9: 868–877. doi:10.1101/gr.9.9.868. PubMed: 10508846. 10508846 PMC310812

[16] Papadopoulos JS, Agarwala R (2007) COBALT: constraint-based alignment tool for multiple protein sequences. Bioinformatics 23: 1073–1079. doi:10.1093/bioinformatics/btm076. PubMed: 17332019. 17332019

[17] Waterhouse AM, Procter JB, Martin DMA, Clamp M, Barton GJ (2009) Jalview version 2 a multiple sequence alignment editor and analysis workbench. Bioinformatics 25: 1189–1191 10.1093/bioinformatics/btp033 PMC2672624 19151095

[18] Tamura K, Dudley J, Nei M, Kumar S (2007) MEGA4: Molecular Evolutionary Genetics Analysis (MEGA) software version 4.0. Mol Biol Evol 24: 1596–1599. doi:10.1093/molbev/msm092. PubMed: 17488738. 17488738

[19] Jones DT, Taylor WR, Thornton JM (1992) The rapid generation of mutation data matrices from protein sequences. Comput Appl Biosci CABIOS 8: 275–282. PubMed: 1633570. 1633570 10.1093/bioinformatics/8.3.275

[20] Cole C, Barber JD, Barton GJ (2008) The Jpred 3 secondary structure prediction server. Nucleic Acids Res 36: W197–W201. doi:10.1093/nar/gkn238. PubMed: 18463136. 18463136 PMC2447793

[21] Krieger E, Vriend G (2002) Elmar Krieger and Gert Vriend. Bioinformatics 18: 315–318. doi:10.1093/bioinformatics/18.2.315. PubMed: 11847079. 11847079

[22] Wiederstein M, Sippl MJ (2007) ProSA-web: interactive web service for the recognition of errors in three-dimensional structures of proteins. Nucleic Acids Res 35: W407–W410. doi:10.1093/nar/gkl865. PubMed: 17517781. 17517781 PMC1933241

[23] Neshich G, Rocchia W, Mancini AL, Yamagishi MEB, Kuser PR et al. (2004) JavaProtein Dossier: a novel web-based data visualization tool for comprehensive analysis of protein structure. Nucleic Acids Res 32: W595–W601. doi:10.1093/nar/gkh118. PubMed: 15215458. 15215458 PMC441618

[24] Neshich G, Mazoni I, Oliveira SRM, Yamagishi MEB, Kuser-Falcão PR et al. (2006) The Star STING server: a multiplatform environment for protein structure analysis. Genet Molecular Res GMR 5: 717–722. 17183482

[25] Konagurthu AS, Whisstock JC, Stuckey PJ, Lesk AM (2006) MUSTANG: a multiple structural alignment algorithm. Proteins 64: 559–574. doi:10.1002/prot.20921. PubMed: 16736488. 16736488

[26] Guex N, Peitsch MC (1997) SWISS-MODEL and the Swiss-PdbViewer: an environment for comparative protein modeling. Electrophoresis 18: 2714–2723. doi:10.1002/elps.1150181505. PubMed: 9504803. 9504803

[27] Neshich G, Mancini AL, Yamagishi MEB, Kuser PR, Fileto R et al. (2005) STING Report: convenient web-based application for graphic and tabular presentations of protein sequence, structure and function descriptors from the STING database. Nucleic Acids Res 33: D269–D274. PubMed: 15608194. 15608194 10.1093/nar/gki111 PMC540065

[28] Mancini AL, Higa RH, Oliveira A, Dominiquini F, Kuser PR et al. (2004) STING Contacts: a web-based application for identification and analysis of amino acid contacts within protein structure and across protein interfaces. Bioinformatics 20: 2145–2147. doi:10.1093/bioinformatics/bth203. PubMed: 15073001. 15073001

[29] Fernandes JH, Hayashi M, Camargo A, Neshich G (2003) Structural basis of the lisinopril-binding specificity in N- and C-domains of human somatic ACE. Biochem Biophys Res Commun 308: 533–539. 10.1016/s0006-291x(03)01363-9 12901857

[30] Marcellino LH, Neshich G, Grossi De Sá MF, Krebbers E, Gander ES (1996) Modified 2S albumins with improved tryptophan content are correctly expressed in transgenic tobacco plants. FEBS Lett 385: 154–158. doi:10.1016/0014-5793(96)00375-4. PubMed: 8647241. 8647241

[31] Ribeiro C, Togawa RC, Neshich IA, Mazoni I, Mancini AL et al. (2010) Analysis of binding properties and specificity through identification of the interface forming residues (IFR) for serine proteases in silico docked to different inhibitors. BMC Struct Biol 10: 36. doi:10.1186/1472-6807-10-36. PubMed: 20961427. 20961427 PMC2974730

[32] Sitkiewicz I, Nagiec MJ, Sumby P, Butler SD, Cywes-Bentley C et al. (2006) Emergence of a bacterial clone with enhanced virulence by acquisition of a phage encoding a secreted phospholipase A2. Proc Natl Acad Sci U S A 103: 16009–16014. doi:10.1073/pnas.0607669103. PubMed: 17043230. 17043230 PMC1635118

[33] Cox GM, McDade HC, Chen SC, Tucker SC, Gottfredsson M et al. (2001) Extracellular phospholipase activity is a virulence factor for *Cryptococcus* *neoformans* . Mol Microbiol 39: 166–175. doi:10.1046/j.1365-2958.2001.02236.x. PubMed: 11123698. 11123698

[34] Korbsrisate S, Tomaras AP, Damnin S, Ckumdee J, Srinon V et al. (2007) Characterization of two distinct phospholipase C enzymes from *Burkholderia* *pseudomallei* . Microbiology 153: 1907–1915. doi:10.1099/mic.0.2006/003004-0. PubMed: 17526847. 17526847

[35] Li X, Gao M, Han X, Tao S, Zheng D et al. (2012) Disruption of the phospholipase D gene attenuates the virulence of *Aspergillus* *fumigatus* . Infect Immun 80: 429–440. doi:10.1128/IAI.05830-11. PubMed: 22083709. 22083709 PMC3255654

[36] Tambourgi DV, Pedrosa MFF, De Andrade RMG, Billington SJ, Griffiths M et al. (2007) Sphingomyelinases D induce direct association of C1q to the erythrocyte membrane causing complement mediated autologous haemolysis. Mol Immunol 44: 576–582. doi:10.1016/j.molimm.2006.02.002. PubMed: 16540172. 16540172

[37] Binford GJ, Bodner MR, Cordes MHJ, Baldwin KL, Rynerson MR et al. (2009) Molecular Evolution, Functional Variation and Proposed Nomenclature of the Gene Family That Includes Sphingomyelinase D in Sicariid Spider Venoms. Mol Biol Evol 26: 547–566. PubMed: 19042943. 19042943 10.1093/molbev/msn274 PMC2767091

[38] Hedayati MT, Pasqualotto a C, Warn P a, Bowyer P, Denning DW (2007) *Aspergillus* *flavus*: human pathogen, allergen and mycotoxin producer. Microbiology (Reading, England) 153: 1677–1692. doi:10.1099/mic.0.2007/007641-0. PubMed: 17526826. 17526826

[39] Health TC for FS and P, Biologics I for IC in A (2005). Dermatophytosis: 1–7.

[40] Dighe NS, Pattan SR, Bhawar SB, Gaware VM (2009). arling ’ s Dis Rev. 1: 88–101.

[41] Ameen M, Talhari C, Talhari S (2010) Advances in paracoccidioidomycosis. Clin Exp Dermatol 35: 576–580. PubMed: 19874328. 19874328 10.1111/j.1365-2230.2009.03647.x

[42] Ampel NM (2010) New perspectives on coccidioidomycosis. Proc Am Thorac Soc 7: 181–185. doi:10.1513/pats.200907-080AL. PubMed: 20463246. 20463246

[43] Bagagli E, Bosco SMG, Theodoro RC, Franco M (2006) Phylogenetic and evolutionary aspects of *Paracoccidioides* *brasiliensis* reveal a long coexistence with animal hosts that explain several biological features of the pathogen. Infect Genet Evol J Mol Epidemiol Evol Genet Infect Dis 6: 344–351. PubMed: 16473563. 10.1016/j.meegid.2005.12.002 16473563

[44] Kiss T, Cadar D, Spînu M (2012) Tick prevention at a crossroad: new and renewed solutions. Vet Parasitol 187: 357–366. doi:10.1016/j.vetpar.2012.02.010. PubMed: 22424918. 22424918

[45] Health TC for FS and P, Biologics I for IC in A (2009). Rhipicephalus appendiculatus: 1–2.

[46] Edwards KT (2011) Gotch ear: a poorly described, local, pathologic condition of livestock associated primarily with the Gulf Coast tick, *Amblyomma* *maculatum* . Vet Parasitol 183: 1–7. doi:10.1016/j.vetpar.2011.09.038. PubMed: 22047764. 22047764

[47] Bramwell PA, Wiener P, Akkermans AD, Wellington EM (1998) Phenotypic, genotypic and pathogenic variation among streptomycetes implicated in common scab disease. Lett Appl Microbiol 27: 255–260. doi:10.1046/j.1472-765X.1998.00439.x. PubMed: 9830140. 9830140

[48] Bazzini S, Udine C, Riccardi G (2011) Molecular approaches to pathogenesis study of *Burkholderia* *cenocepacia*, an important cystic fibrosis opportunistic bacterium. Appl Microbiol Biotechnol 92: 887–895. doi:10.1007/s00253-011-3616-5. PubMed: 21997606. 21997606

[49] Jurgenson JE, Zeller KA, Leslie JF (2002) Expanded Genetic Map of *Gibberella* *moniliformis* (*Fusarium* *verticillioides*). Appl Environ Microbiol 68: 1972–1979. doi:10.1128/AEM.68.4.1972-1979.2002. PubMed: 11916720. 11916720 PMC123879

[50] Albermann S, Linnemannstöns P, Tudzynski B (2013) Strategies for strain improvement in *Fusarium* *fujikuroi*: overexpression and localization of key enzymes of the isoprenoid pathway and their impact on gibberellin biosynthesis. Appl Microbiol Biotechnol 97: 2979–2995. doi:10.1007/s00253-012-4377-5. PubMed: 22983595. 22983595

[51] Thomma BPHJ, Esse Hpvan, Crous PW, Wit PJGMDE (2005) Pathogen profile *Cladosporium* *fulvum* ( syn . *Passalora* *fulva* ), a highly specialized plant pathogen as a model for functional studies on plant pathogenic Mycosphaerellaceae. Mol Plant Pathol 6: 379–393. doi:10.1111/j.1364-3703.2005.00292.x. PubMed: 20565665. 20565665

[52] Selvy PE, Lavieri RR, Lindsley CW, Brown HA (2011) Phospholiapse D - enzymology, functionality and chemical modulation. Chem Rev 12; 111(10): 6064–6119. 10.1021/cr200296t PMC3233269 21936578

[53] Van Meeteren LA, Frederiks F, Giepmans BNG, Pedrosa MFF, Billington SJ et al. (2004) Spider and bacterial sphingomyelinases D target cellular lysophosphatidic acid receptors by hydrolyzing lysophosphatidylcholine. J Biol Chem 279: 10833–10836. doi:10.1074/jbc.C300563200. PubMed: 14732720. 14732720

[54] Horta CCR, Oliveira-Mendes BBR, Ao do Carmo, Siqueira, Barroca FF et al. (2013) Lysophosphatidic acid mediates the release of cytokines and chemokines by human fibroblasts treated with loxosceles spider venom. J Invest Dermatol 6: 1682–1685. PubMed: 23353984. 10.1038/jid.2013.40 23353984

