# Supplementary material for: Identification of New Sphingomyelinases D in Pathogenic Fungi and Other Pathogenic Organisms
Source: PLoS One. 2013 Nov 1;8(11):e79240. doi: 10.1371/journal.pone.0079240 (PMC3815110; doi:10.1371/journal.pone.0079240)
Supplement: Figure S1 — Analysis of the contact characteristics of the SMS-tail residues in the 3D structure of the L. laeta SMase D (1xx1.pdb) according to STING predicted contacts. The carbon atoms for the stick-represented residues are colored based on the STING color codes for the contact types: green for salt bridges or a charge attractive interaction, red for a charge repulsive interaction, gray for aromatic stacking, purple for a hydrophobic interaction, light pink for hydrogen bonds involving the main chain, orange for hydrogen bonds involving the main chain and a side chain and white for the query/central residue. The contact partners and interaction type are shown for the SMS-tail residues in the PDB file (1xx1.pdb): Arg271 (A), Leu272 (B), Ala273 (C), Thr274 (D), Asp277 (E), Pro279 (F) and Trp280 (G). An overall view of the SMS-tail is shown (H). STING graphical contact representation for the most important residues in terms of contact energy: Asp277 (I) and Trp280 (J). (DOCX) [file pone.0079240.s001.docx]

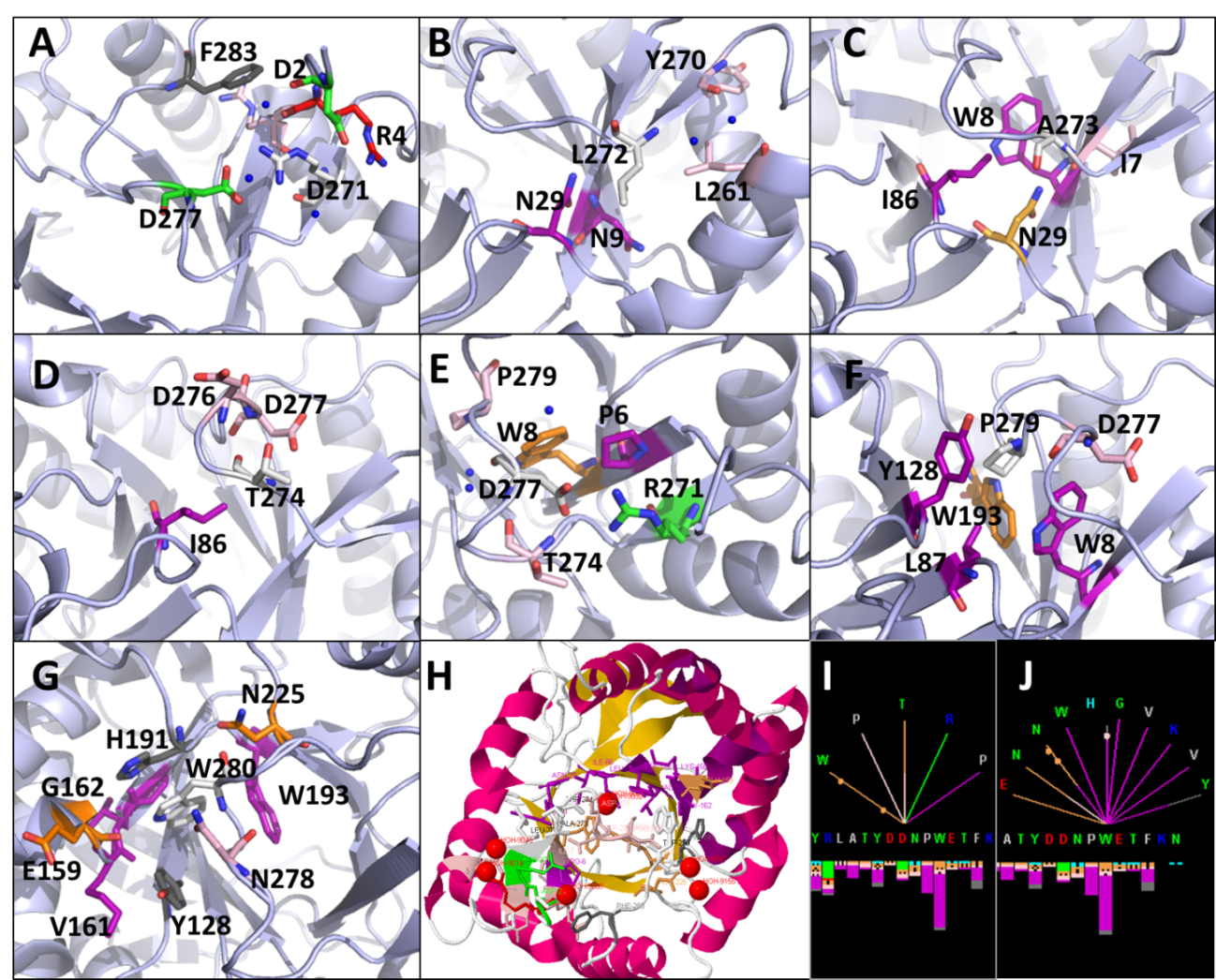


**Figure S1**: Analysis of the contact characteristics of the SMS-tail residues in the 3D structure of the *L. laeta* SMase D (1xx1.pdb) according to STING predicted contacts. The carbon atoms for the stick-represented residues are colored based on the STING color codes for the contact types: green for salt bridges or a charge attractive interaction, red for a charge repulsive interaction, gray for aromatic stacking, purple for a hydrophobic interaction, light pink for hydrogen bonds involving the main chain, orange for hydrogen bonds involving the main chain and a side chain and white for the query/central residue. The contact partners and interaction type are shown for the SMS-tail residues in the PDB file (1xx1.pdb): Arg271 (A), Leu272 (B), Ala273 (C), Thr274 (D), Asp277 (E), Pro279 (F) and Trp280 (G). An overall view of the SMS-tail is shown (H). STING graphical contact representation for the most important residues in terms of contact energy: Asp277 (I) and Trp280 (J).
