# Supplementary material for: Identification of New Sphingomyelinases D in Pathogenic Fungi and Other Pathogenic Organisms
Source: PLoS One. 2013 Nov 1;8(11):e79240. doi: 10.1371/journal.pone.0079240 (PMC3815110; doi:10.1371/journal.pone.0079240)
Supplement: Table S1 — Bacterial species found to contain an SMaseD, with the corresponding databases for the sequences indicated. (DOCX) [file pone.0079240.s002.docx]

Table S1: Bacterial species found to contain an SMaseD, with the corresponding databases for the sequences indicated.

| **Kingdom** | **Phylum** | **Order** | **Family** | Bacterial species found to contain a similar SMaseD sequence | **Number of entries found in NCBI databases** | | | |
| --- | --- | --- | --- | --- | --- | --- | --- | --- |
|  |  |  |  |  | **Protein nr** | **dbEST** | **WGS** | **TSA** |
| Bacteria | Actinobacteria | Actinomycetales | Dermato-philaceae | *Austwickia chelonae NBRC 105200* | **0** | **0** | **2** | **0** |
|  |  |  | Actino-mycineae | *Arcanobacterium haemolyticum* | **3** | **0** | **0** | **0** |
|  |  |  |  | *Arcanobacterium haemolyticum DSM 20595* | **12** | **0** | **0** | **0** |
|  |  |  | Corynebacteriaceae | *Corynebacterium pseudotuberculosis* | **9** | **0** | **0** | **0** |
|  |  |  |  | *Corynebacterium pseudotuberculosis 1/06-A* | **6** | **0** | **0** | **0** |
|  |  |  |  | *Corynebacterium pseudotuberculosis 1002* | **6** | **0** | **0** | **0** |
|  |  |  |  | *Corynebacterium pseudotuberculosis 258* | **6** | **0** | **0** | **0** |
|  |  |  |  | *Corynebacterium pseudotuberculosis 267* | **6** | **0** | **0** | **0** |
|  |  |  |  | *Corynebacterium pseudotuberculosis 3/99-5* | **6** | **0** | **0** | **0** |
|  |  |  |  | *Corynebacterium pseudotuberculosis 31* | **6** | **0** | **0** | **0** |
|  |  |  |  | *Corynebacterium pseudotuberculosis 316* | **6** | **0** | **0** | **0** |
|  |  |  |  | *Corynebacterium pseudotuberculosis 42/02-A* | **6** | **0** | **0** | **0** |
|  |  |  |  | *Corynebacterium pseudotuberculosis C231* | **6** | **0** | **0** | **0** |
|  |  |  |  | *Corynebacterium pseudotuberculosis CIP 52 97* | **6** | **0** | **0** | **0** |
|  |  |  |  | *Corynebacterium pseudotuberculosis Cp162* | **6** | **0** | **0** | **0** |
|  |  |  |  | *Corynebacterium pseudotuberculosis FRC41* | **6** | **0** | **0** | **0** |
|  |  |  |  | *Corynebacterium pseudotuberculosis I19* | **6** | **0** | **0** | **0** |
|  |  |  |  | *Corynebacterium pseudotuberculosis P54B96* | **6** | **0** | **0** | **0** |
|  |  |  |  | *Corynebacterium pseudotuberculosis PAT10* | **6** | **0** | **0** | **0** |
|  |  |  |  | *Corynebacterium ulcerans* | **21** | **0** | **0** | **0** |
|  |  |  |  | *Corynebacterium ulcerans 0102* | **9** | **0** | **0** | **0** |
|  |  |  |  | *Corynebacterium ulcerans 809* | **6** | **0** | **0** | **0** |
|  |  |  |  | *Corynebacterium ulcerans BR-AD22* | **6** | **0** | **0** | **0** |
|  |  |  | Streptomyce-taceae | *Streptomyces lysosuperficius ATCC 31396* | **0** | **0** | **1** | **0** |
|  |  |  |  | *Streptomyces sp. AA1529* | **0** | **0** | **1** | **0** |
|  |  |  |  | *Streptomyces sp. Mg1* | **6** | **0** | **1** | **0** |
|  | Proteo-bacteria | Burkhol-deriales | Burkhol-deriaceae | *Burkholderia cenocepacia HI2424* | **2** | **0** | **0** | **0** |
