# Supplementary material for: Identification of New Sphingomyelinases D in Pathogenic Fungi and Other Pathogenic Organisms
Source: PLoS One. 2013 Nov 1;8(11):e79240. doi: 10.1371/journal.pone.0079240 (PMC3815110; doi:10.1371/journal.pone.0079240)
Supplement: Table S2 — Arachnid species found to contain an SMaseD, with the corresponding database sources for the sequences indicated. (DOCX) [file pone.0079240.s003.docx]

Table S2: Arachnid species found to contain an SMaseD, with the corresponding database sources for the sequences indicated.

| **Kingdom** | **Phylum** | **Order** | **Family** | Arachnid species found to contain a similar SMaseD sequence | **Number of entries found in NCBI databases** | | | |
| --- | --- | --- | --- | --- | --- | --- | --- | --- |
|  |  |  |  |  | **Protein nr** | **dbEST** | **WGS** | **TSA** |
| Metazoa | Arthropoda, Chelicerata | Araneae, Mygalomorphae | Theraphosidae | *Acanthoscurria gomesiana* | **0** | **3** | **0** | **0** |
|  |  | Araneae, Araneomorphae | Sicariidae | *Loxosceles adelaida* | **2** | **0** | **0** | **0** |
|  |  |  |  | *Loxosceles aff. spinulosa GJB-2008* | **8** | **0** | **0** | **0** |
|  |  |  |  | *Loxosceles amazonica* | **16** | **0** | **0** | **0** |
|  |  |  |  | *Loxosceles apachea* | **42** | **0** | **0** | **0** |
|  |  |  |  | *Loxosceles arizonica* | **61** | **0** | **0** | **0** |
|  |  |  |  | *Loxosceles boneti* | **9** | **0** | **0** | **0** |
|  |  |  |  | *Loxosceles cf. spinulosa GJB-2008* | **4** | **0** | **0** | **0** |
|  |  |  |  | *Loxosceles deserta* | **69** | **0** | **0** | **0** |
|  |  |  |  | *Loxosceles gaucho* | **5** | **0** | **0** | **0** |
|  |  |  |  | *Loxosceles hirsuta* | **110** | **0** | **0** | **0** |
|  |  |  |  | *Loxosceles intermedia* | **68** | **228** | **0** | **0** |
|  |  |  |  | *Loxosceles laeta* | **78** | **127** | **0** | **0** |
|  |  |  |  | *Loxosceles reclusa* | **12** | **0** | **0** | **0** |
|  |  |  |  | *Loxosceles rufescens* | **24** | **0** | **0** | **0** |
|  |  |  |  | *Loxosceles sabina* | **48** | **0** | **0** | **0** |
|  |  |  |  | *Loxosceles similis* | **4** | **0** | **0** | **0** |
|  |  |  |  | *Loxosceles sp 4 GJB-20* | **4** | **0** | **0** | **0** |
|  |  |  |  | *Loxosceles sp. 4 GJB-2008* | **8** | **0** | **0** | **0** |
|  |  |  |  | *Loxosceles spadicea* | **44** | **0** | **0** | **0** |
|  |  |  |  | *Loxosceles spinulosa* | **48** | **0** | **0** | **0** |
|  |  |  |  | *Loxosceles variegata* | **36** | **0** | **0** | **0** |
|  |  |  |  | *Sicarius albospinosus* | **4** | **0** | **0** | **0** |
|  |  |  |  | *Sicarius cf. damarensis GJB-2008* | **84** | **0** | **0** | **0** |
|  |  |  |  | *Sicarius patagonicus* | **16** | **0** | **0** | **0** |
|  |  |  |  | *Sicarius peruensis* | **68** | **0** | **0** | **0** |
|  |  |  |  | *Sicarius terrosus* | **8** | **0** | **0** | **0** |
|  |  |  | Eresidae | *Stegodyphus lineatus* | **0** | **0** | **0** | **1** |
|  |  |  |  | *Stegodyphus mimosarum* | **0** | **0** | **0** | **1** |
|  |  |  |  | *Stegodyphus tentoriicola* | **0** | **0** | **0** | **3** |
|  |  | Ixodida | Ixodidae | *Amblyomma maculatum* | **12** | **0** | **0** | **6** |
|  |  |  |  | *Rhipicephalus appendiculattus* | **0** | **10** | **0** | **0** |
|  |  |  |  | *Rhipicephalus microplus* | **0** | **19** | **0** | **0** |
|  |  |  |  | *Dermatophagoides pteronyssinus* | **0** | **2** | **0** | **0** |
|  |  |  |  | *Ixodes scapularis* | **18** | **18** | **14** | **0** |
|  |  | Mesostigmata | Phytoseiidae | *Metaseiulus occidentalis* | **0** | **0** | **4** | **2** |
|  |  |  | Varroidae | *Varroa destructor* | **0** | **0** | **3** | **0** |
|  |  | Astigmata | Psoroptidae | *Psoroptes ovis* | **0** | **1** | **0** | **0** |
|  |  |  | Pyroglyphidae | *Dermatophagoides pteronyssinus* | **0** | **2** | **0** | **0** |
|  |  | Prostigmata | Tetranychidae | *Tetranychus urticae* | **0** | **9** | **10** | **1** |
