# Supplementary material for: Identification of New Sphingomyelinases D in Pathogenic Fungi and Other Pathogenic Organisms
Source: PLoS One. 2013 Nov 1;8(11):e79240. doi: 10.1371/journal.pone.0079240 (PMC3815110; doi:10.1371/journal.pone.0079240)
Supplement: Table S3 — Fungal species found to contain an SMaseD, with the corresponding database sources for the sequences indicated. (DOCX) [file pone.0079240.s004.docx]

Table S3: Fungal species found to contain an SMaseD, with the corresponding database sources for the sequences indicated.

| **Kingdom** | **Phylum** | **Order** | **Family** | Fungal species found to contain a similar SMaseD sequence | **Number of entries found in NCBI databases** | | | |
| --- | --- | --- | --- | --- | --- | --- | --- | --- |
|  |  |  |  |  | **Protein nr** | **dbEST** | **WGS** | **TSA** |
| Fungi | Ascomycota | Onygenales | Ajellomycetaceae | *Ajellomyces capsulatus G186AR* | **3** | **0** | **1** | **0** |
|  |  |  |  | *Ajellomyces capsulatus G217B* | **0** | **0** | **1** | **0** |
|  |  |  |  | *Ajellomyces capsulatus H143* | **3** | **0** | **1** | **0** |
|  |  |  |  | *Ajellomyces capsulatus H88* | **3** | **0** | **1** | **0** |
|  |  |  |  | *Ajellomyces capsulatus NAm1* | **6** | **0** | **1** | **0** |
|  |  |  |  | *Ajellomyces dermatitidis ATCC 18187* | **0** | **0** | **1** | **0** |
|  |  |  |  | *Ajellomyces dermatitidis ATCC 18188* | **3** | **0** | **1** | **0** |
|  |  |  |  | *Ajellomyces dermatitidis ATCC 26199* | **0** | **0** | **1** | **0** |
|  |  |  |  | *Ajellomyces dermatitidis ER-3* | **3** | **0** | **1** | **0** |
|  |  |  |  | *Ajellomyces dermatitidis SLH14081* | **6** | **0** | **0** | **0** |
|  |  |  |  | *Histoplasma capsulatum var. capsulatum Tmu* | **0** | **0** | **1** | **0** |
|  |  |  | Arthrodermataceae | *Arthroderma benhamiae CBS 112371* | **6** | **0** | **1** | **0** |
|  |  |  |  | *Trichophyton equinum CBS 127.97* | **3** | **0** | **1** | **0** |
|  |  |  |  | *Trichophyton rubrum CBS 118892* | **6** | **3** | **1** | **0** |
|  |  |  |  | *Trichophyton tonsurans CBS 112818* | **3** | **0** | **1** | **0** |
|  |  |  |  | *Trichophyton verrucosum HKI 0517* | **6** | **0** | **1** | **0** |
|  |  |  | Onygenaceae | *Uncinocarpus reesii 1704* | **6** | **0** | **1** | **0** |
|  |  |  | mitosporic Onygenales | *Coccidioides immitis H538.4* | **0** | **0** | **2** | **0** |
|  |  |  |  | *Coccidioides immitis RMSCC 3703* | **0** | **0** | **4** | **0** |
|  |  |  |  | *Coccidioides immitis RS* | **12** | **21** | **2** | **0** |
|  |  |  |  | *Coccidioides posadasii C735* | **12** | **0** | **2** | **0** |
|  |  |  |  | *Coccidioides posadasii CPA 0001* | **0** | **0** | **2** | **0** |
|  |  |  |  | *Coccidioides posadasii CPA 0020* | **0** | **0** | **2** | **0** |
|  |  |  |  | *Coccidioides posadasii CPA 0066* | **0** | **0** | **2** | **0** |
|  |  |  |  | *Coccidioides posadasii RMSCC 1037* | **0** | **0** | **3** | **0** |
|  |  |  |  | *Coccidioides posadasii RMSCC 1038* | **0** | **0** | **2** | **0** |
|  |  |  |  | *Coccidioides posadasii RMSCC 2133* | **0** | **0** | **2** | **0** |
|  |  |  |  | *Coccidioides posadasii RMSCC 2394* | **0** | **0** | **2** | **0** |
|  |  |  |  | *Coccidioides posadasii RMSCC 3488* | **0** | **0** | **2** | **0** |
|  |  |  |  | *Coccidioides posadasii RMSCC 3700* | **0** | **0** | **2** | **0** |
|  |  |  |  | *Coccidioides posadasii RMSCC 3703* | **0** | **0** | **4** | **0** |
|  |  |  |  | *Coccidioides posadasii str. Silveira* | **6** | **49** | **2** | **0** |
|  |  |  |  | *Paracoccidioides brasiliensis Pb03* | **3** | **0** | **1** | **0** |
|  |  |  |  | *Paracoccidioides brasiliensis Pb18* | **3** | **0** | **2** | **0** |
|  |  |  |  | *Paracoccidioides sp. 'lutzii' Pb01* | **6** | **0** | **1** | **0** |
|  |  | Eurotiales | Trichocomaceae | *Aspergillus flavus NRRL3357* | **18** | **0** | **3** | **0** |
|  |  |  |  | *Aspergillus oryzae 3.042* | **9** | **6** | **3** | **0** |
|  |  |  |  | *Aspergillus oryzae AS 3.863* | **0** | **0** | **3** | **0** |
|  |  |  |  | *Aspergillus oryzae AS 3.951* | **0** | **0** | **3** | **0** |
|  |  |  |  | *Aspergillus oryzae RIB326* | **0** | **0** | **3** | **0** |
|  |  |  |  | *Aspergillus oryzae RIB40* | **18** | **0** | **0** | **0** |
|  |  | Hypocreales | Nectriaceae | *Gibberella moniliformis 7600* | **0** | **0** | **1** | **0** |
|  |  |  |  | *Fusarium fujikuroi B14* | **0** | **0** | **2** | **0** |
|  |  |  |  | *Fusarium oxysporum f. sp lycopersici 4287* | **0** | **0** | **1** | **0** |
|  |  |  |  | *Fusarium oxysporum f. sp. melonis 26406* | **0** | **0** | **1** | **0** |
|  |  |  | Clavicipitaceae | *Metarhizium acridum CQMa 102* | **6** | **0** | **2** | **0** |
|  |  |  |  | *Metarhizium anisopliae ARSEF 23* | **12** | **0** | **5** | **0** |
|  |  | Capnodiales | Mycosphaerellaceae | *Passalora fulva CBS 131901* | **0** | **0** | **1** | **0** |
